# Comparing watershed black locust afforestation and natural revegetation impacts on soil nitrogen on the Loess Plateau of China

**DOI:** 10.1038/srep25048

**Published:** 2016-04-26

**Authors:** Zhao Jin, Xiangru Li, Yunqiang Wang, Yi Wang, Kaibo Wang, Buli Cui

**Affiliations:** 1 State Key Laboratory of Loess and Quaternary Geology, Institute of Earth Environment, Chinese Academy of Sciences, Xi’an 710061, China; 2 Shaanxi Key Laboratory of Accelerator Mass Spectrometry Technology and Application, Xi’an AMS Center, Xi’an 710061, China; 3 School of Resources and Environmental Engineering, Ludong University, Yantai 264025, China

## Abstract

This study examined a pair of neighbouring small watersheds with contrasting vegetations: artificial forestland and natural grassland. Since 1954, afforestation which mainly planted with black locust has been conducted in one of these watersheds and natural revegetation in the other. The differences in soil total N, nitrate, ammonium, foliar litterfall δ^15^N and dual stable isotopes of δ^15^N and δ^18^O in soil nitrate were investigated in the two ecosystems. Results showed that there was no significant difference in soil total N storage between the two ecosystems, but the black locust forestland presented higher soil nitrate than the grassland. Moreover, the foliar litterfall N content and δ^15^N of the forestland were significant higher than the grassland. These results indicate that 60 years of watershed black locust afforestation have increased soil N availability. The higher nitrate in the forestland was attributed to the biological N fixation of black locust and difference in ecosystem hydrology. The dual stable isotopes of δ^15^N and δ^18^O revealed that the two ecosystems had different sources of soil nitrate. The soil nitrate in the forestland was likely derived from soil N nitrification, while the soil nitrate in the grassland was probably derived from the legacy of NO_3_^−^ fertiliser.

Afforestation, which is the conversion of historically treeless areas into forests, is the most important forestry practice contributing to vegetation restoration and ecosystem rehabilitation. In humid regions, afforestation is thought to be appropriate for promoting ecosystem restoration and carbon sequestration. However, in arid and semiarid regions, this method has raised grave concerns^1^,^2^,^3^. Numerous studies have demonstrated that dryland afforestation can exacerbate water shortages and lead to the deterioration of soil ecosystems in many cases^4^,^5^,^6^. In addition to water depletion, dryland afforestation could also consume large amounts of soil nutrients, especially nitrogen (N)^7^,^8^. Previous studies have demonstrated that afforestation or forest regrowth can deplete soil N pools^9^,^10^ and/or decrease soil N availability^11^,^12^,^13^. In drylands, available soil N is low^14^,^15^; thus, progressive N limitation would be more serious as a result of afforestation. However, the extent to which soil N availability will change during drylands afforestation remains poorly understood. A better understanding of these processes will assist in assessing the potential of soil N limitation in forest ecosystem construction.

An alternative to afforestation that also promotes ecosystem restoration and carbon sequestration is the protection and facilitation of natural revegetation on abandoned lands^16^,^17^. In contrast to afforestation, natural revegetation represents a natural process of recovering ecosystem function and can therefore be used as a control for the study of ecosystem biogeochemical cycles. Exploring the differences in soil N storage and availability between natural and artificial measures can provide information that is valuable for the prediction of changes in soil N cycling. Studies by Zhang *et al*.^18^,^19^ demonstrated that natural revegetation in humid areas improves soil N nutrient levels to a greater extent than tree plantation. Moreover, Li *et al*.^13^ found that cropland afforestation in humid areas could lead to progressive N limitation. Drylands occupy 47 percent of the surface of the earth^20^. However, information on the effects of the natural and artificial management practices on soil N in drylands is still limited. Moreover, few studies have used the dual stable isotopes of δ^15^N and δ^18^O to characterise the changes in soil N cycling during drylands afforestation or natural revegetation.

Recently, several studies have demonstrated that foliar δ^15^N is a useful indicator of ecosystem N availability^21^,^22^,^23^. The basic premise is that δ^15^N measured in organic material characterises the fractionation processes^24^,^25^. When the N supply is high relative to biotic demand, N is lost through fractionating pathways; the remaining ecosystem N is enriched in ^15^N^26^. Therefore, ecosystems with high N availability typically exhibit high δ^15^N values in plant tissues^27^. Moreover, dual stable isotopes (δ^15^N and δ^18^O) of nitrate have been used successfully to identify the sources and transformation processes of nitrate in water and terrestrial ecosystems^28^,^29^,^30^,^31^. In many cases, the dual stable isotopes offer a direct means of source identification because different sources of nitrate often have different isotopic compositions^32^. For example, nitrate and ammonium fertilisers, animal and human wastes, and soils have distinct δ^15^N and δ^18^O levels, which can be used to distinguish the sources of nitrate^32^.

The Loess Plateau of China is a unique geographical area that is characterised by an extensive loess distribution, severe soil erosion and low vegetation coverage. Since the 1950s, the Chinese government has made great efforts to control soil erosion and restore vegetation, including implementing large-scale tree plantation in the 1970s, integrated soil erosion control in the 1980s and 1990s and the Grain for Green Project in the 2000s^33^,^34^. The most recent study by Lü *et al*.^35^ demonstrated that a total of 8.69 × 10^5^ ha of cropland was converted to forestland on the Loess Plateau between 2000 and 2008. This extensive afforestation has been reported to decrease regional water yield and deplete soil water resources^35^,^36^. However, it is unknown whether several decades of afforestation will lead to more serious N limitation along with soil water deficits. The resulting information will greatly assist in understanding the effects of dryland afforestation on soil N cycling.

In this study, a pair of neighbouring small watersheds with similar topographical and geological backgrounds on the Loess Plateau were selected and used to compare the effects of artificial affestatation and natural revegetation on soil N storage and availability (Fig. 1). Since 1954, afforestation which mainly planted with black locust has been conducted in one of these watersheds and natural revegetation in the other. The two watersheds have formed completely different ecosystems: black locust forestland and natural grassland. In a previous study, we found that the two ecosystems have different patterns of soil carbon cycling and the grassland is more beneficial to soil surface organic and inorganic carbon sequestration than the forestland^37^. The objectives of this study were (1) to examine the difference in soil N storage and availability between the two ecosystems, and (2) to use the dual stable isotopes of δ^15^N and δ^18^O to identify the soil nitrate source.

## Results

### Soil physical and chemical properties in the two ecosystems

Significant differences were identified in the physical and chemical properties of the soils in the two ecosystems (Table 1). The black locust forestland had higher soil bulk density and lower soil organic carbon (SOC), soil moisture and C/N compared with the natural grassland. However, no significant difference between the two ecosystems could be identified with respect to the soil pH and total N concentrations (STN).

### Soil total N, nitrate and ammonium content in the two ecosystems

**Figure 2:**
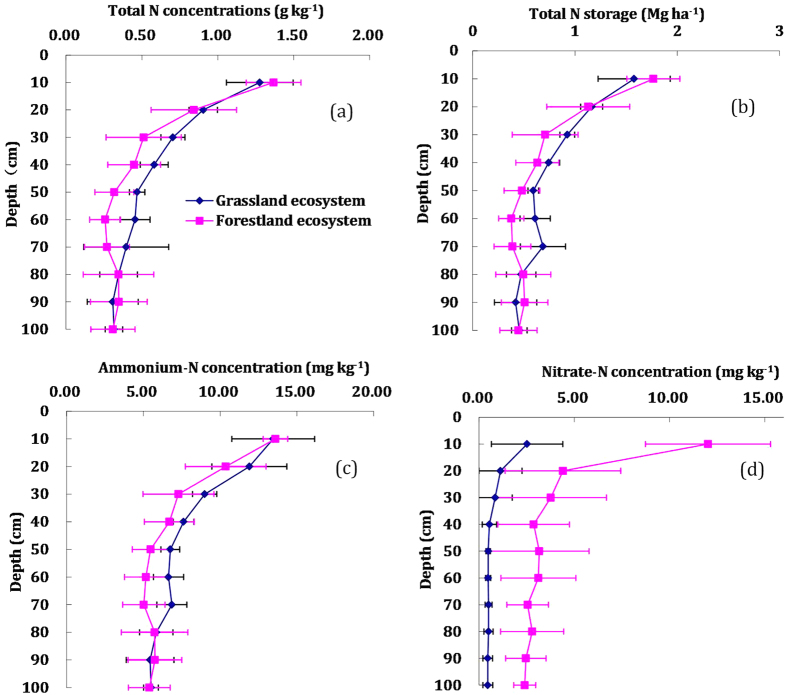
Contents of soil total N, nitrate and ammonium between the forestland and grassland ecosystems.

The soil total N content exhibited no significant difference between the forestland and grassland ecosystems (Fig. 2a,b, P = 0.702). The storages of soil total N down to a depth of 1 m were 6.93 and 7.65 Mg ha^−1^ for the black locust forestland and natural grassland, respectively. The concentrations of soil nitrate were significantly different between the two ecosystems, with the content of soil nitrate being significantly higher in the black locust forestland than in the natural grassland (Fig. 2d, *P* = 0.003), with mean values at a depth of 1 m of 3.95 and 0.79 mg kg^−1^, respectively. Moreover, the soil ammonium content exhibited no significant difference between the forestland and grassland ecosystems (Fig. 2c, *P* = 0.499).

### Foliar N content and natural abundance of ^15^N in the two ecosystems

**Figure 3:**
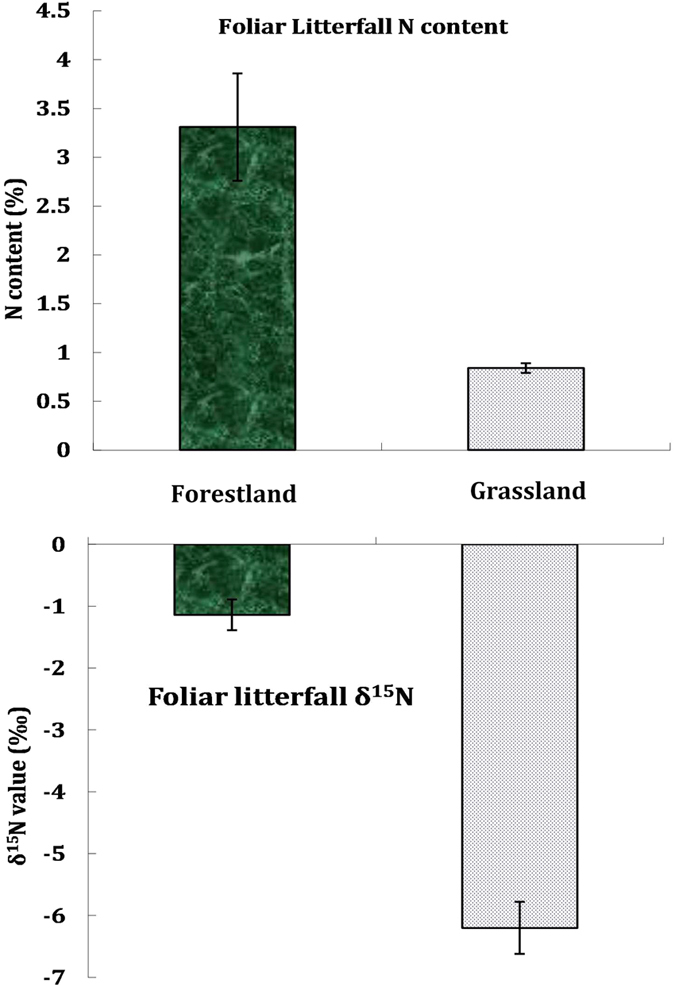
Foliar litterfall N content and δ^15^N between the forestland and grassland ecosystems.

In this study, the black locust forestland exhibited a higher foliar litterfall N content and δ^15^N than the natural grassland (Fig. 3, *P* < 0.0001). The mean foliar N content and δ^15^N in the black locust forestland were 3.31% and -1.14‰, respectively, and 0.84% and −6.20‰, respectively, for the natural grassland.

### Natural abundance of soil nitrate ^15^N and ^18^O in the two ecosystems

**Figure 4:**
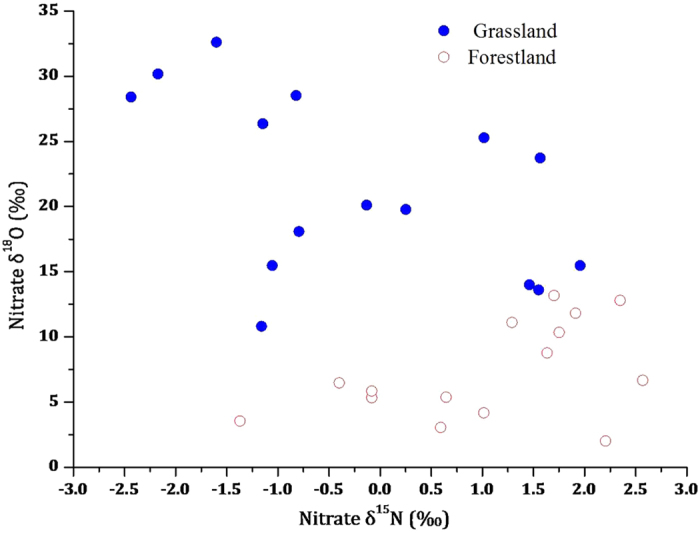
Dual stable isotopes of δ^15^N and δ^18^O in soil nitrate between the forestland and grassland ecosystems.

In this study, soil nitrate δ^15^N and δ^18^O also exhibited a large difference between the forestland and grassland ecosystems (Fig. 4). The nitrate δ^15^N level ranged from −1.37‰ to 2.57‰ in the black locust forestland and from −2.44‰ to 1.96‰ in the natural grassland. The δ^18^O level ranged from 3.05‰ to 13.16‰ in the black locust forestland and from 10.80‰ to 32.61‰ in the natural grassland.

## Discussion

In this study, the artificial forestland and natural grassland provides a unique opportunity to examine the effects of long-term dryland afforestation and natural revegetation on soil N storage and availability. Our results showed that the two ecosystems exhibited no significant difference in soil total N storage (Fig. 2b), while the content of soil nitrate in the black locust forestland was significantly higher than in the natural grassland (Fig. 2d). Davidson *et al*.^21^,^38^,^39^ demonstrated that higher nitrate in ecosystems typically indicates excess available N relative to plant demand and higher soil N availability. Rather than decreasing soil N availability, the higher nitrate content of the black locust forestland investigated in this study indicates that 60 years of watershed black locust afforestation have increased soil N availability. Moreover, the results of the foliar litterfall δ^15^N analysis demonstrated that the black locust forestland had a higher foliar N content and δ^15^N level than the natural grassland (Fig. 3), which further proved that the forestland had higher soil N availability than the grassland.

In a previous study, Jiao *et al*.^6^ assessed the ecological success of restoration by afforestation on the northern Loess Plateau and found that afforestation with black locust offered few additional advantages when compared with natural recovery sites. Moreover, Wei *et al*.^40^ showed that the conversion of grasslands to pine woodlands on the northern Loess Plateau reduced soil N availability. However, Liu *et al*.^41^ evaluated the ecological functions of black locust on the Loess Plateau and found that black locust had the potential to improve soil N availability. Besides, Qiu *et al*.^42^ demonstrated that afforestation with black locust in loessial gully region of the Loess Plateau obviously improved soil N levels and the improvements were greater in long-term than middle-term black locust stands. In this study, we conclude that two factors have led to the increased soil N availability in the forestland. One factor is the excess N input due to biological N fixation of black locust. In the forestland, the main planted species is black locust, which is well known to be a symbiotic N fixer. Previous studies have demonstrated that black locust has high rates of biological N fixation^43^,^44^. Lopez *et al*.^45^ showed that approximately 10 years after black locust establishment, soil N had already been enriched by black locust N. In this study, the plantation age is approximately 60 years; thus, the forestland N has probably been enriched by black locust N. Therefore, the biological N fixation by black locust could be the dominant factor contributing to the increased soil N availability in the forestland. The second factor is the difference in ecosystem hydrology. Many studies have demonstrated that the two ecosystems exhibit a large difference in hydrology^46^,^47^. The annual runoff in the forestland was observed to be significantly lower than in the grassland^37^,^48^,^49^. Moreover, the soil water content of the forestland was lower than in the grassland, and soil desiccation had clearly occurred in the forestland due to higher forest transpiration and canopy interception^37^,^46^,^47^. The poor soil water conditions and low runoff have probably decreased the soil nitrate loss and improved the nitrate accumulation in the forestland.

Kendall *et al*.^32^ reviewed that different sources of N had different ranges of δ^15^N_NO3_ and δ^18^O_NO3_ values and therefore could be isotopically distinguishable. Regarding atmospheric N, the δ^15^N values of atmospheric NO_3_^−^ and NH_4_^+^ are usually in the range of −15 to 15‰^32^. Moreover, the δ^18^O_NO3_ values of precipitation surveyed in the late 1990s range from 14 to 75‰^50^, while the δ^18^O_NO3_ values in precipitation across the USA reported more recently ranged from +63 to +94‰, with a mean value of +76.3‰^51^. About inorganic fertilisers, the element of N derives from atmospheric N_2_ and therefore the δ^15^N values are usually low, generally in the range of −4 to +4‰^32^. The synthetic fertilisers, where the O is mostly derived from atmospheric O_2_ (ca. +23.5‰), have δ^18^O values ranging from +17 to +25‰^32^ Moreover, nitrate derived from nitrification of ammonium fertilisers has lower δ^18^O values, usually in the range of −5 to +15‰^32^. About soils, the δ^15^N of total soil N ranges from about −10 to +15‰^32^. Soluble dissolved inorganic nitrogen (mainly NO_3_^−^) represents a very small pool while it is more important than the larger organic pool to maintain ecosystem N availability. The δ^15^N of soil nitrate ranges from about −10 to +15‰, with most soils having δ^15^N_NO3_ values in the range of +2 to +5‰^32^,^50^.

**Figure 5:**
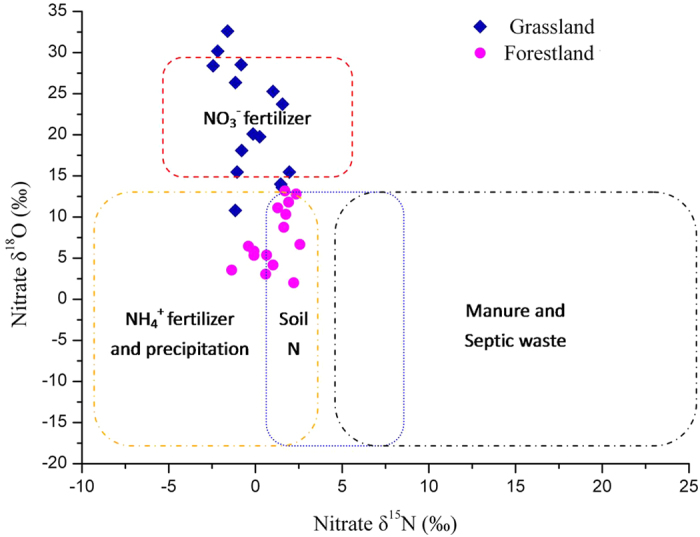
Scatter diagram of δ^15^N versus δ^18^O of nitrate in the soil samples at the ecosystems of forestland and grassland. The figure has not been directly taken from Kendall *et al*.^32^, which was compiled using their published data of the ranges of δ^15^N and δ^18^O values of potential nitrate sources from Kendall *et al*.^32^.

In this study, the δ^15^N and δ^18^O levels in soil nitrate suggested that the two ecosystems were influenced by different sources of nitrate. The nitrate δ^15^N levels in the forestland and grassland were nearly zero. However, the soil nitrate was more enriched with heavy oxygen isotopes in the grassland than in the forestland (Fig. 4). In comparison with the typical nitrate δ^15^N and δ^18^O levels derived or nitrified from various N sources^32^, we found that the soil nitrate of the grassland was more influenced by NO_3_^−^ fertilisers because δ^18^O_NO3_ values of the grassland falls in the range of δ^18^O values of NO_3_^−^ fertiliser (Fig. 5). However, the soil nitrate of the forestland was more influenced by soil N transformation and nitrification (Fig. 5). In the forestland, most of the excessive N derives from the biological N fixation of black locust; thus, the nitrate δ^15^N and δ^18^O signals represent the characteristics of soil N transformation and nitrification; while in the grassland, the dual stable isotopes are indicative of NO_3_^−^ fertiliser, suggesting that NO_3_^−^ fertilisers had been applied to the grassland or the former farmland.

## Materials and Methods

### Study site

This study was conducted in the Nanxiaohe Basin, which is located in the Xifeng District of Qingyang city, Gansu province. The region has a semi-arid continental climate; the mean annual temperature and precipitation are 9.3 °C and 556.5 mm, respectively. Approximately 67.3% of the annual precipitation occurs from June to September. The area has a loess gully landscape with elevations varying from 1,050 m to 1,423 m. The soil layer is approximately 250 m thick, and the soil is silt-loamy^52^.

In the basin, a pair of neighbouring small watersheds with similar topographical and geological backgrounds, the Dongzhuanggou (DZG) watershed and Yangjiagou (YJG) watershed, were selected for this study. DZG is 1.6 km long and covers an area of 1.15 km^2^. Since 1954, DZG has been subject to natural revegetation measures and currently supports grassland vegetation; the principal grass species are *Arundinella hirta, Agropyron cristatum* and *Artemisia argyi*. YJG is 1.5 km long and covers an area of 0.87 km^2^. The principal afforestation activities in YJG occurred from 1954 to 1958; the current timber volume is 4,000 m^3^ 52. The principal planted species is black locust (*Robinia pseudoacacia* L.). Following 60 years of vegetation restoration, the two small watersheds have formed completely different vegetation landscapes (YJG: artificial forestland; DZG: natural grassland).

### Soil sampling and laboratory analysis

Soil sampling was performed in May and September of 2013. To determine the average content and vertical distribution of soil total N (STN), 14 sampling sites were established in the forestland watershed and 14 in the grassland watershed. In the two watersheds, the area of gully slopes occupies more than 65% of the total area of the watersheds^49^ and the measures of black locust afforestation and natural revegetation have been mainly conducted in the area. Therefore, the sampling sites were randomly distributed on the gully slopes, which could represent the pure ecosystems of black locust forestland and natural grassland. In the study, soil samples were collected to a depth of 1 m. The soils were sampled at intervals of 10 cm using a hand-held auger (6 cm in diameter), and 10 soil samples were obtained at each site. Accordingly, 140 soil samples were collected in the forestland watershed and 140 in the grassland watershed. To determine the soil nitrate and ammonium contents, bulk density and pH, three soil profiles at a depth of 0–100 cm were established in each of the forestland and grassland watersheds. For the soil bulk density analysis, three replicate samples were collected at intervals of 10 cm for each profile using a soil corer (a stainless steel cylinder with a volume of 100 cm^3^). Soil samples were collected at the same distance intervals and used to analyse the nitrate and ammonium contents, pH and the dual stable isotopes of nitrate δ^15^N and δ^18^O.

All of the collected soil samples were air-dried in the laboratory; gravel and roots were carefully removed from the soil. The air-dried soil samples were ground in an agate mortar and passed through a 0.15 mm sieve. Soil total N contents were measured through micro-Kjeldahl digestion, followed by distillation and titration^53^. Moreover, SOC was determined using soil samples digested in K_2_Cr_2_O_7_-H_2_SO_4_ solution using a heated oil bath, and the organic carbon concentration was subsequently determined via titration^53^. Soil nitrate and ammonium were extracted with a 2 M KCl solution (soil:solution, 1:5) and filtered through a 0.45 μm filter^13^. A portion of each solution was prepared to determine the nitrate and ammonium concentrations, while the other portion was prepared to determine the nitrate δ^15^N and δ^18^O levels. The nitrate and ammonium concentrations were analysed using a continuous flow analyser (Skalar San++ System, Skalar Analytical B.V., Netherlands). The soil samples used for the bulk density analysis were dried at 115 °C for 24 h. STN storage (Mg ha^−1^) values were calculated as follows:


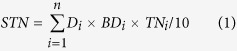


where *D*_*i*_, *BD*_*i*_ and *TN*_*i*_ represent the soil thickness (cm), bulk density (g cm^−3^) and soil total N content (g kg^−1^), respectively, for the *i*th level of the soil profile.

### Foliar δ^15^N and nitrate δ^15^N and δ^18^O analyses

The fresh foliar litterfall was sampled in the forestland and grassland in September 5–14, 2013 and returned to the laboratory. Dust and soil were carefully removed from the surfaces of the foliar samples. The samples were air-dried and ground to a powder. The foliar N content and δ^15^N were analysed using a Vario PYRO cube element analyser and an EA-IsoPrime100 stable isotope ratio mass spectrometer (Isoprime Ltd, U.K). The soil nitrate δ^15^N and δ^18^O levels were prepared via quantitative bacterial reduction of nitrate to nitrous oxide. Nitrous oxide was extracted and purified using a trace gas pre-concentrator unit; the product was analysed using an EA-IsoPrime100 stable isotope ratio mass spectrometer (Yue *et al*., 2014). Three international materials (USGS-32, USGS-34 and USGS-35) were used to calibrate the measured sample data. Each sample was measured in duplicate, and the standard error was 0.3% for nitrate δ^15^N and 0.4% for nitrate δ^18^O.

The isotope ratios (δ^15^N and δ^18^O) are expressed in δ notation as parts per thousand deviations (‰):


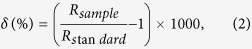


where *R* = ^15^ N/^14^N or ^18^O/^16^O. The ^15^N/^14^N reference is N_2_ in air, whereas the ^18^O/^16^O reference is Vienna standard mean ocean water (VSMOW).

### Statistical analysis

An independent-sample *t*-test was performed to test the significance of the soil property differences, soil N storage and availability at an alpha level of 0.05 (*a* = 0.05) between the forestland and grassland. All statistical analyses were performed with the Statistical Program for Social Sciences (SPSS 11.0, SPSS Inc., 2001).

## Additional Information

**How to cite this article**: Jin, Z. *et al*. Comparing watershed black locust afforestation and natural revegetation impacts on soil nitrogen on the Loess Plateau of China. *Sci. Rep.* **6**, 25048; doi: 10.1038/srep25048 (2016).

## Figures and Tables

**Figure 1:**
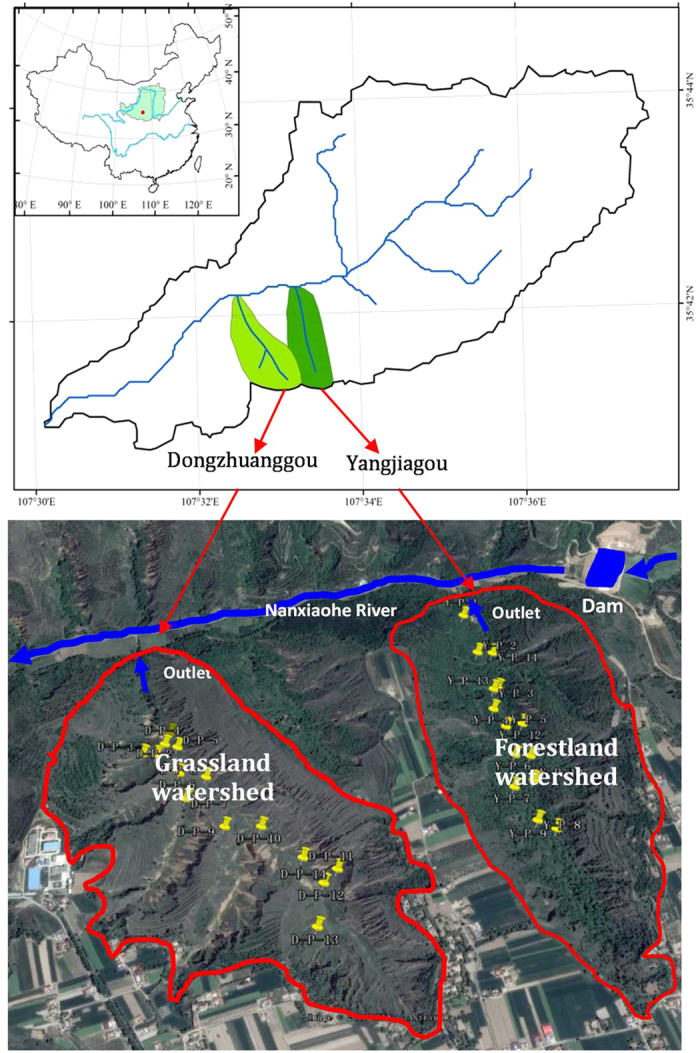
Map showing the location of the study area in Xifeng District, Qingyang city, Gansu province, including the watersheds of forestland and grassland (Chinese map in the figure was created by ArcGIS 9.3 software, http://www.arcgis.com/features/; satellite image in the figure derives from Google Earth and copyright of the satellite image belongs to Google Earth and CNES/Astrium).

**Table 1:** Soil chemical and physical properties between the ecosystems of forestland and grassland.

Soil properties	Grassland	Forestland
SOC (g kg^−1^)	5.22 ± 0.83^a^	3.84 ± 1.28^b^
STN (g kg^−1^)	0.58 ± 0.13^a^	0.50 ± 0.18^a^
C/N ratio	9.05 ± 2.27^a^	7.68 ± 1.24^b^
pH	8.42 ± 0.10^a^	8.44 ± 0.08^a^
Soil bulk density (g cm^−3^)	1.34 ± 0.07^a^	1.44 ± 0.11^b^
Soil moisture (%)*	19.1%^a^	13.4%^b^

Notes: *Soil moisture data was extracted from Wang *et al*.^47^; group designated by the same letter are not significantly different at *p* < 0.05.
